# P06 Stewardship potential—the hidden burden of antibiotic prophylaxis in primary care

**DOI:** 10.1093/jacamr/dlad077.010

**Published:** 2023-08-02

**Authors:** Carol Philip, Alexander Fullbrook, Simon Dewar, Morgan Evans, Donald Inverarity, Meghan Perry, Michelle Etherson

**Affiliations:** Clinical Infection Research Group (CIRG), NHS Lothian Infection Service, Edinburgh, UK; Clinical Infection Research Group (CIRG), NHS Lothian Infection Service, Edinburgh, UK; Clinical Infection Research Group (CIRG), NHS Lothian Infection Service, Edinburgh, UK; Clinical Infection Research Group (CIRG), NHS Lothian Infection Service, Edinburgh, UK; Clinical Infection Research Group (CIRG), NHS Lothian Infection Service, Edinburgh, UK; Clinical Infection Research Group (CIRG), NHS Lothian Infection Service, Edinburgh, UK; Clinical Infection Research Group (CIRG), NHS Lothian Infection Service, Edinburgh, UK

## Abstract

**Background:**

This study aimed to decrease unnecessary long-term antimicrobial use in primary care by identifying and highlighting patients with long-term or repeated exposure to antibiotics to GP teams for review. The broad spectrum antimicrobial co-amoxiclav was targeted following data showing rising DDD per prescription in our Health Board.

**Methods:**

Community antibiotic prescribing from the national Prescribing Information System (PIS) was searched for NHS Lothian (126 GP practices) using DDD to identify patients. Long courses of co-amoxiclav were defined as ≥63 DDD. Ninety-eight patients were identified with high antimicrobial usage and a data collection form was sent to the Primary Care Pharmacy teams to voluntarily collate data for each patient.

**Results:**

The data collection form had a 97% response rate. Of the 94 patients with full data 62 (66%) patients had co-amoxiclav initiated due to secondary care specialist advice and 26 (28%) had been initiated by primary care. Forty-four patients (47%) were aged 65 or over, putting them at high risk of *Clostridioides difficile*. Forty-one patients had multiple courses of co-amoxiclav and 39 patients were prescribed co-amoxiclav as prophylaxis; of these 59% were for urinary tract infection (UTI). Thirty-four of 94 patients (36%) had been on prophylaxis for over 5 years with 9 patients on co-amoxiclav for over 20 years. Of note in 39 of 94 patients no microbiology samples were available and where samples were available (*n*=55), narrower spectrum options were available in 38 patients (69%) and 17 patients (31%) had samples showing resistance to co-amoxiclav. Following this intervention to highlight use, the number of patients on long-term courses or prophylaxis with co-amoxiclav reduced by 70% in the following year.

**Conclusions:**

Patients on long-term antibiotics are priorities for antimicrobial stewardship interventions. This work demonstrated that ongoing appropriateness and efficacy of long-term antibiotic courses were not routinely reviewed. Using pharmacy data proved an effective method to highlight patients and ultimately reduce patient antibiotic exposure. UTI prophylaxis was the majority patient group in this study and interventions to review and decrease long-term UTI prophylaxis in our Board using this method are underway.

